# Mediastinal Mature Cystic Teratoma With Intrapulmonary Extension Mimicking a Hydatid Cyst: Advantages of MRI in Diagnostics

**DOI:** 10.7759/cureus.57745

**Published:** 2024-04-06

**Authors:** Andrić Viktorija, Dajana Tuksar, Primož Caf

**Affiliations:** 1 Radiology, University Medical Centre Maribor, Maribor, SVN

**Keywords:** extragonadal germ cell tumor, lipoid pneumonia, mature teratoma, pulmonary hydatid cyst, mediastinal cystic mass

## Abstract

We report the case of a 37-year-old male patient, who presented with a chief complaint of a sudden throbbing pain in the left side of the chest. Imaging techniques revealed a cystic mass in the anterior mediastinum and the left upper lung lobe. Despite a high suspicion of a hydatid cyst due to the clinical history of the patient and the cystic nature of the lesion, CT and subsequent MRI confirmed the presence of a cystic teratoma, entailing surgical intervention for removal. If untreated, a teratoma can cause significant and life-threatening complications.

## Introduction

Mature cystic teratomas are rare germ-cell tumors that primarily originate in the gonads. The anterior mediastinum is the most common extragonadal site of germ cell tumors, accounting for 15% of all mediastinal tumors, of which 50% to 70% are teratomas [1]. At extragonadal sites, most of the teratomas are mature type and benign, but approximately 1-3% of cases may contain immature elements and are either overtly malignant or undergo a malignant transformation [1-3]. Diagnostics of a cystic teratoma can sometimes be difficult, as they can mimic other mediastinal cystic masses, such as a hydatid cyst in our case [4]. However, several specific findings help us distinguish them on imaging modalities, including the location and MRI appearance, as we will discuss later in this article. Since teratomas have unpredictable natural progression and potentially life-threatening complications, complete surgical resection is the primary treatment of choice [1].

## Case presentation

**Figure 1:**
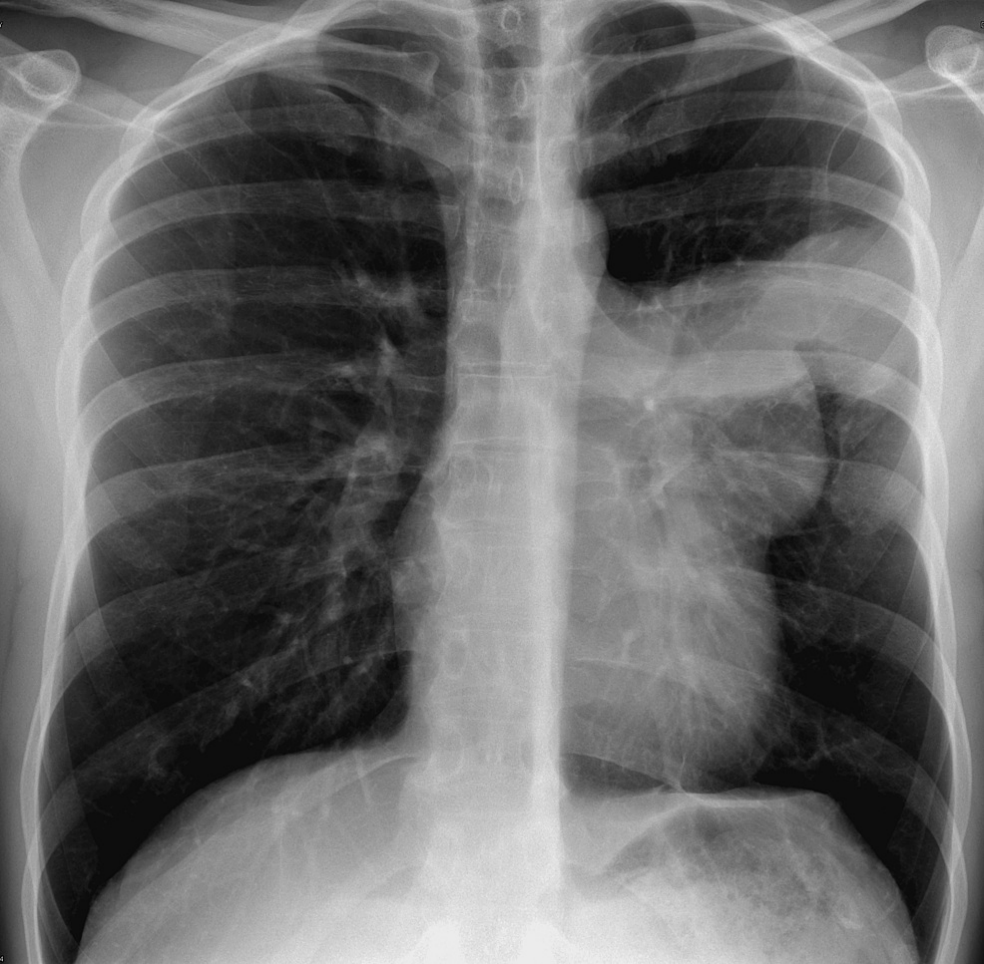
Chest X-ray showing a well-marginated mass in the left perihilar region.

A 37-year-old patient was admitted to the psychiatric department due to social problems, which are in part related to excessive alcohol consumption. During hospitalization, he reported a rapid onset of throbbing pain in the left side of the chest. Initially, acute myocardial infarction was ruled out by performing ECG and laboratory tests. Subsequently, a chest X-ray revealed a well-circumscribed mass in the left perihilar region, raising suspicion of an inflammatory infiltrate due to elevated inflammatory markers (Figure 1). This led to the administration of antibiotics for the treatment of pneumonia.

**Figure 2:**
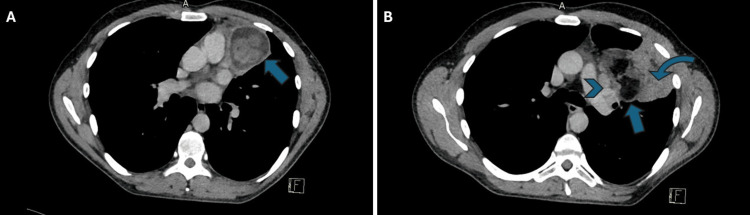
Axial chest computed tomography (CT) images showing mediastinal mass. The lower part of the mass is represented in image (A), which displays a round structure that appears to be cystic (arrow). The upper part of the mass is shown in image (B), which displays elements of fat (arrow), enlarged mediastinal lymph nodes (arrowhead), and consolidated lung tissue (curved arrow).

Following the 12th day of antibiotic therapy and repeated X-rays with no signs of improvement, the patient underwent a chest CT scan with IV contrast, which revealed a larger, heterogeneous mediastinal mass. The mass contained various components, like soft tissue, fat, and fluid. It was situated along the anterior mediastinum and pericardium on the left side, extending from the hilum to the anterior and lateral thoracic wall. The mass was compressing the surrounding structures, especially the left upper lobe, and could not be properly delineated from the surrounding tissue (Figure 2). These findings were suggestive of a mediastinal teratoma, although due to a substantial cystic component diagnostics was challenging, such that a hydatid cyst was included among the differential diagnoses.

Given serious potential complications that arise in the setting of surgical excision of hydatid cysts, attempts were made to decrease a pre-test probability of this condition. The patient underwent a serology test for *Echinococcus granulosus*, which was negative. However, it is imperative to note that serology tests alone may not be sufficient to exclude the possibility of an infection. Furthermore, levels of beta-hCG, alpha-fetoprotein, and AChR antibodies were ascertained to address the differential of a teratoma and a thymoma. All tests came back with results that were within normal limits.

**Figure 3:**
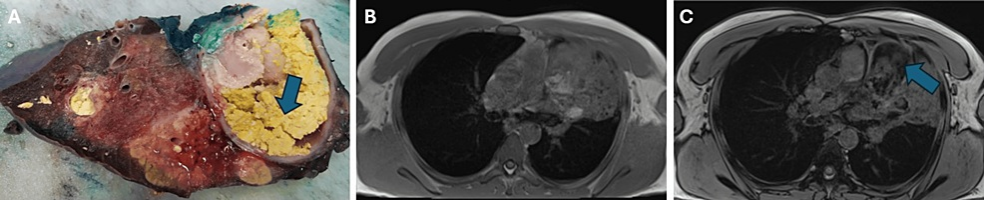
Macroscopic appearance and magnetic resonance imaging (MRI) of the mediastinal teratoma. Teratoma is represented in image (A) (obtained from the Department of Pathology), which displays cysts and sebaceous material components (arrow). Axial T1 in-phase (B) and opposed-phase images (C) showing hypointensity areas in T1 opposed-phase image in the sebaceous material (arrow).

Consequently, an MRI of the chest with IV contrast was conducted, revealing a heterogeneous mass, consisting of several different tissues. Most of the mass consisted of a cystic component that appeared hyperintense on T2-weighted images. As on the CT examination, the lateral part had a large consolidation with areas of the same intensity in some parts of the neighboring left upper lobe. The central part presented as a T2 and T1 hyperintense lesion, with a significant drop in signal intensity on fat saturation weighting. Moreover, on the T1 opposed phase, an even greater drop in signal was seen in the sebaceous material. All described signal changes were indicative of the presence of fat or fatty material. These MRI findings strongly suggested the presence of a teratoma (Figure 3).

**Figure 4:**
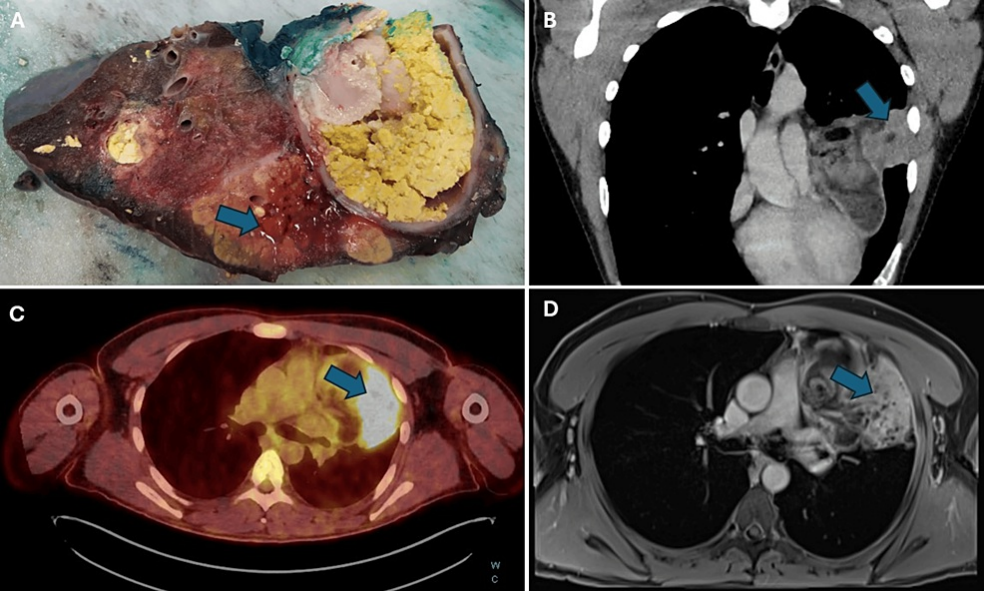
Macroscopic appearance and radiographic features of the lipoid pneumonia. There is a macroscopic display of signs of pneumonia (arrow) in the lung tissue with some fatty elements in the adjacent area (A). A retrospective analysis of the paracoronal multiplanar reconstruction (B) displayed communication between the teratoma and the lung (arrow). PET CT scan showed high metabolic activity in the lateral part of the mass which was indicative of lipoid pneumonia retrospectively (C). MRI T1 post-contrast pictures (D) showing pneumonia with some fatty elements in the bronchial tree (arrow).

In addition, a PET-CT scan was performed, which revealed the presence of a mass in the anterior mediastinum spreading to the left upper lobe. The metabolic activity of the lateral part of the mass indicated the possibility of malignancy, although no differential diagnosis was provided. Upon retrospective analysis, it was determined that the high metabolic activity was due to lipoid pneumonia (Figure 4). Furthermore, the PET CT scan results indicated the presence of three lymph nodes suspicious for potential regional lymph node metastasis, although later pathologic examination of the lymph nodes did not reveal any malignancy.

After a comprehensive diagnostic evaluation, the patient underwent a surgical removal of the mass and the left upper lobe. The surgical findings revealed that the tumor occupied a considerable portion of the left upper lung lobe and was locally invasive.

Upon macroscopic examination, the margins of the resected tumor showed complete removal of the lesion with a safety margin. Further pathological analysis of the excised mass confirmed the diagnosis of mature cystic teratoma. The examination also revealed extensive histological features of lipoid pneumonia in the neighboring left upper lobe, likely resulting from overgrowth and communication within the tracheobronchial tree.

## Discussion

Generally, teratomas are classified as mature or immature based on their differentiation status. Mature teratomas are mostly benign with rare malignant transformation. Immature teratomas are more likely to be malignant and found exclusively in male patients [3].

Patients with teratomas are frequently asymptomatic for a long time, and the tumor is found incidentally on chest X-ray. Most of the symptoms are related to compression of adjacent structures and include chest pain, dyspnea, cough, fever, and fatigue [1,5,6]. Other symptoms can be presented in the event of rupture, which is a rare but potentially serious complication. Rupture or overgrowth into the tracheobronchial tree can cause hemoptysis and trichoptysis or recurrent pulmonary infection or inflammatory disease as observed in our case of lipoid pneumonia. Teratomas can also rupture into the pleural or pericardial cavity, leading to pleural effusion or cardiac tamponade [3,6,7]. In addition, superior vena cava syndrome or brachial plexus neuritis can occur because of pericardial or vascular ingrowth of the mass [1].

A chest X-ray is usually the initial investigation, which often reveals an anterior mediastinal mass [1]. In our case, an initial chest X-ray was performed to exclude other possible causes of acute chest pain, revealing a large mass in the left hilar region.

A chest CT scan with an IV contrast is usually the preferred method of investigation. Nonetheless, the results may occasionally demonstrate non-specific findings. In cases where malignancy is suspected, further studies, such as an MRI of the chest or PET-CT, may be required to establish a definitive diagnosis [5,6,8].

An MRI is a more reliable method than CT for distinguishing between cystic and solid lesions. Cystic components show homogeneous hyperintensity on T2-weighted images but variable intensity on T1-weighted images. On the other hand, soft tissue elements typically exhibit isointesity with muscle characteristics. MRI has the added advantage of detecting both macroscopic and microscopic intracellular fat, while CT can only determine the presence of macroscopic fat. Macroscopic fat on MRI appears as an area of high signal intensity on T1- or T2-weighted images. Moreover, if a fat-fluid level is observable within an anterior mediastinal mass, it is pathognomonic of a teratoma, indicating the presence of sebum. In addition, in- and opposed-phase chemical shift gradient echo imaging provides further insights into the existence of microscopic or intravoxel fat in teratomas [5,9-11].

The initial preoperative diagnosis in our case was in favor of a mature teratoma, along with the possibility of a hydatid cyst, due to the patient history of alcoholism and potentially unhealthy lifestyle habits. Hydatid cyst disease is a zoonotic infection transmitted via the fecal-oral route. Pulmonary hydatid cysts typically affect the lower lobes, with a preference for the right basal lobe. Cystic teratomas and hydatid cysts may mimic each other and distinguishing between the two requires careful interpretation of the patient’s clinical context, serological data, and radiographic imaging, with CT and MRI being the most useful modalities for detecting pulmonary cysts and their complications [4,8,12]. In our case, MRI was the most accurate imaging modality, showing elements of fat and other characteristics of cystic teratoma, such as intraparenchymal infiltration and direct bronchial connection, which is a distinguishing feature of intrapulmonary teratoma.

The differential diagnosis of a mediastinal cystic mass in the anterior mediastinum is extensive, and often several entities must be considered, such as thymomas, lymphomas, lymphangiomas, hydatid cyst, and other non-neoplastic cystic lesions, a mediastinal or lung abscesses, and of course teratoma (as in our case) or other germ-cell tumors [1,8,13].

An infiltrating anterior mediastinal cystic lesion with a high suspicion of malignancy usually demands a complete surgical excision [6]. Our patient underwent an open thoracotomy resection without preoperative histologic confirmation, as in other cases in the literature. This clinical management decision was made because surgery is a treatment of choice and since we excluded the presence of hydatid cyst radiologically with the proof of elements of fat on MRI, which all pointed towards the diagnosis of teratoma.

Definitive diagnosis hinges on a comprehensive surgical pathology examination of the definitive surgical excision. The specimen should be sampled exhaustively, to exclude any malignant component and immature tissue elements. Within the gonads, many teratomas that show chromosomal abnormalities, specifically 12p gains or isochromosome 12p (i12p), carry a higher risk of malignant potential even if the sampled material consists entirely of mature teratoma only. At extragonadal sites, ancillary testing is not well-studied and not typically employed, but the largest recent case series at an extragonadal anatomic site showed a negative i12p status, regardless of mature or immature teratoma component, and all patients showed favorable outcomes [14].

## Conclusions

Mediastinal mature teratomas are rare tumors, which are usually asymptomatic and frequently detected incidentally on chest X-ray or CT scan of the chest. The differential diagnosis of mediastinal cystic lesions at first can be extensive, occasionally leading to initial misdiagnosis, hence highlighting the importance of proper imaging. Chest CT and MRI are highly sensitive investigations when interpreted by an experienced radiologist, which frequently lead to an accurate diagnosis of mediastinal cystic teratoma and successfully exclude the presence of a hydatid cyst or other cystic masses. Despite the benign nature of these tumors and generally good long-term prognosis, complete surgical resection remains the treatment of choice. This is advised to prevent possible life-threatening complications, such as rupture or ingrowth of the mass in adjacent structures, or the presence of a malignant component. Long-term follow-up is recommended to detect any potential recurrence, although it is uncommon.
